# Enhanced Adsorption of Tetracycline by Thermal Modification of Coconut Shell-Based Activated Carbon

**DOI:** 10.3390/ijerph192113741

**Published:** 2022-10-22

**Authors:** Do-Gun Kim, Shinnee Boldbaatar, Seok-Oh Ko

**Affiliations:** 1 Department of Environmental Engineering, Sunchon National University, 255 Jungang-ro, Suncheon 57922, Korea; 2 Department of Civil Engineering, Kyung Hee University, 1732, Deakyungdaero, Yongin 17104, Korea

**Keywords:** activated carbon, adsorption, antibiotic, thermal modification, thermodynamics

## Abstract

Tetracycline (TC) is one of the most frequently detected antibiotics in various water matrices, posing adverse effects on aquatic ecosystems. In this study, coconut shell-based powdered activated carbon (PAC) was thermally modified under various temperatures to enhance TC adsorption. The PAC subjected to 800 °C (PAC800) showed the best TC adsorption. PAC and PAC800 were characterized using N_2_ adsorption/desorption isotherm, X-ray photoelectron spectroscopy, Raman spectroscopy, XRD, Boehm titration, and zeta potential analyses. Increases in the specific surface area, C/O ratio, C=O, surface charge, basic groups, and the number of stacked graphene layers along with a decrease in structural defects were observed for PAC800 compared to PAC. The TC adsorption was significantly improved for PAC800 compared to that of PAC, which is attributable to the enhanced electrostatic attraction and π-π EDA interactions induced by the changes in the properties. The Freundlich isotherm was the best fit indicating the heterogeneous nature, and the Freundlich constant of PAC and PAC800 increased from 85.8 to 119.5 and 132.1 to 178.6 (mg/g)‧(L/mg)^1/n^, respectively, when the temperature was increased from 296.15 to 318.15 K. The kinetics were well described by the pseudo-second-order adsorption model and the rate constant of PAC and PAC800 increased from 0.80 to 1.59 and from 0.72 to 1.29 × 10^−3^ g/mg‧min, respectively, as the temperature was increased. The activation energy of PAC and PAC800 was 23.7 and 19.6 J/mol, respectively, while the adsorption enthalpy was 196.7 and 98.5 kJ/mol, respectively, indicating endothermic nature. However, it was suggested that TC adsorption onto PAC800 was more favorable and was more contributed to by physisorption than that onto PAC. These results strongly suggest that the properties, adsorption capacity, and adsorption mechanisms of carbonaceous adsorbents can be significantly changed by simple thermal treatment. More, the results provide valuable information about the design of carbonaceous adsorbents with better performance where the structures and functional groups, which positively affect the adsorption, must be improved.

## 1. Introduction

Tetracycline (TC) is a member of the TC family along with chlortetracycline (CTC), oxytetracycline (OTC), and doxycycline (DC), which are the most widely used antibiotics because of their variety of applications, high quality, and low cost [1]. TC is highly water soluble, hydrophilic with a low octanol-water partition coefficient (Kow) of 1.25, and relatively stable in acidic media with low pKa values (Table S1) [2,3]. TC is not readily metabolized and up to 75% of the dosed amount is excreted [4], resulting in increases in the TC concentration in the environment with increasing manure application [5,6].

The released TC is transported to the aquatic environment through various pathways in the hydrogeologic cycle [5,7]. Therefore, TC is one of the most detected pharmaceuticals in natural waters and wastewaters [8], resulting in adverse effects on aquatic ecosystems, including changes in the microbial community structure as well as the promotion of antibiotic-resistant bacteria and antibiotic resistance genes [5]. The detected concentrations of TC were up to 110,000 ng/L in rivers such as the Yangtze River and Wenyu River, China; 1450 ng/L in lakes including Chaohu Lake, China, and Lake Victoria, Uganda; 2500 ng/L in seawaters collected in the Yellow Sea, China, Northern coastline of the Iranian Persian Gulf, Iran, and others; 29,700 ng/L in groundwaters in Beijing, China, agricultural areas in Germany, and others; 632 ng/L in drinking water sources in Lower reaches of the Yangtze River, China, and Huaihe River Basin, China; 2860 μg/L in livestock wastewaters in Jiangsu, China, Shanghai, China, and others; 12.34 μg/L in wastewaters in Beijing, China, The United States, and others; 156,000 ng/g in sediments the Persian Gulf, Iran, Changhshou Lake, China, and others; and 1620 μg/kg in sludges in South China, Brazil, and others [1]. It was reported that the growth of *M. aeruginosa* was inhibited most significantly by TC, where the EC10 values were 0.63, 1.86, and 3.02 mg/L for TC, CTC, and OTC, respectively [9]. Therefore, TC is more toxic than CTC and OTC.

In this regard, a variety of technologies have been attempted for the effective removal of TC from aqueous media, such as chlorination, ozonation, photocatalysis, ionic exchange, biological treatment, and membranes. However, most of them have drawbacks, such as high cost, incomplete oxidation and the generation of toxic byproducts, significant effects of co-existing ions on performance, and poor biodegradability [7,10,11]. On the other hand, it is regarded that adsorption is more efficient than these technologies because of its benefits such as simple application, easy operation, no byproduct formation, and cost-effectiveness [3,7,10].

A variety of adsorbents have been investigated for TC removals, such as hydrous ferric oxide, biochar, activated carbon (AC), α-Fe_2_O_3_/reduced graphene oxide, metal–organic frameworks (MOFs), MnO_2_/biochar, and Fe_3_O_4_/graphene [3]. Among them, AC is the most widely studied and applied for the removal of organic compounds in water treatment [12]. AC is prepared from various materials such as wood, coal, and coconut shells, which result in different TC adsorption amounts. Huang et al. [8] and Son et al. [13] demonstrated that the adsorption rate and capacity of TC and/or OTC were in the order of coal-, coconut shell-, and wood-based AC.

Though coconut shell-based AC has a lower TC adsorption capacity than coal-based AC, it is the most widely used type of AC worldwide because of the cheap and abundant raw material, i.e., coconut shells [14]. Coconut shell is one of the coconut wastes along with husk, frond, fiber, and pulp, and it produced 62.5 million tons per year [15,16]. It is produced mainly in coconut-producing countries in tropical areas, such as Indonesia, Malaysia, Philippines, Hawaii, Africa, South America, and the Pacific islands [17]. Coconut is produced in 92 countries, but 75% of it is from Asian countries [18]. The weight of the coconut shell accounts for approximately 85% of that of the fruit [19]. It seems clear that coconut shell is one of the most abundant waste biomass in dozens of countries, however, it is difficult to quantify the amount of it because of inappropriate disposal [20]. It is regarded that coconut shell is one of the ideal raw materials for ACs because of its abundant supply with the economic feasibility of manufacture, good natural structure for preparing porous materials, and low ash content [21,22]. Therefore, the demand for coconut shell-based ACs is increasing in the Asia Pacific region, which is also the major supplier of coconut shells, with increasing requirements for water quality management [23]. Coconut shell-based ACs are widely used for the removal of a variety of water pollutants, such as residual organic matter (OM) of secondary wastewater treatment and portable water treatment, natural OM, pharmaceuticals, endocrine disrupting chemicals, disinfection byproducts, and the OMs causing odor and tastes, because of abundant functional groups and high specific surface area [21,22].

Therefore, the improvement of the performance of coconut shell-based AC may be one of the best alternatives in the removal of organic micropollutants including TC. Many modification methods have been evaluated to improve the adsorption capacity of ACs such as thermal treatment, chemical functionalization, hetero-atom doping, and composites with metal (hydro)oxides [3,24]. Among them, the thermal treatment of carbonaceous materials is known to be simple and avoids chemical waste [24]. There are many recent publications about the effect of carbonization and/or activation temperature, during the preparation of adsorbents, on adsorption [25,26,27]. However, there is a limited number of reports about the effects of thermal treatment of manufactured ACs. Some of them are about the enhanced acetaminophen adsorption by abiochar after the treatment at 750 °C [28], improved adsorption of phenol, 4-chlorophenol, and 2,4-dichlorophenol by an AC via thermal treatment at 1500–1800 °C [24], and enhanced phosphorus adsorption by an AC after it was treated at 1073–1373 K [29]. On the other hand, previous studies have shown that multi-stage activation during production improved the property of ACs and their performance. The enhanced specific surface area and pore volume, compared to single-stage activation, was reported for the two-stage activation of anthracite by heat and KOH [30], of *Acer truncatum* seed shells by KOH and heat [31], of brewer’s spent grains by H_3_PO_4_ and KOH [32], of the cotton stalk by KOH and heat [33], and of soybean stalk by KOH and heat [34]. These also suggest the potential of the additional thermal treatment of already prepared ACs to improve adsorption.

Though there are encouraging reports about the enhanced adsorption of thermally treated carbonaceous materials, it is hard to find the details of what changes induced by thermal treatment would lead to improved adsorption [24,28,29,35,36]. The adsorption characteristics of a carbon-based adsorbent are known to be dependent not only on the surface area but also on the functional groups and the properties of graphitic structures [28,37] since the adsorption of organic compounds on them is largely attributed to electrostatic attraction, hydrophobic interaction, and π-π electron donor-acceptor (EDA) interactions [38]. In this regard, the changes in the properties of an AC through thermal treatment would provide valuable information about the improvement of the performance of carbon-based adsorbents in organic micropollutant removal.

Therefore, in this study, a coconut shell-based AC was thermally modified under various temperatures. The ACs were characterized regarding their pore structure and surface properties. Then, the kinetics and the equilibrium of TC adsorption on the ACs were investigated to identify the key characteristic changes induced by the thermal treatment.

## 2. Materials and Methods

### 2.1. Chemicals and Materials

TC hydrochloride (C_22_H_24_N_2_O_8_·HCl, TC·HCl), hydrochloric acid (HCl, 37%), sodium carbonate (Na_2_CO_3_, 99.95–100.05%), sodium bicarbonate (NaHCO_3_, 99.5–100.5%), and sodium hydroxide (NaOH, ≥97%) were purchased from Merck KGaA (Darmstadt, Germany). All were of analytical grade and used as received. Distilled, deionized water (DDIW) was obtained using an Aquapuri 551 system (Younglin, Korea). A coconut shell-based powdered AC (PAC) was purchased from Samchully Activated Carbon Co, Ltd. (Seoul, Korea), washed with 1% (*w*/*w*) HCl, and then with DDIW until the pH was 7.0 ± 0.2. The PAC was dried overnight at 150 °C and then heated at a ramp of 5 °C/min, and maintained for 2 h at 500, 600, 700, 800, and 900 °C in a tube furnace (GSL-1600X-50-UL, MTI, USA) under an Ar flow of 50 mL/min. The PAC treated at 500, 600, 700, 800, and 900 °C was denoted PAC500, PAC600, PAC700, PAC800, and PAC900, respectively.

### 2.2. Characterization

The pore structure and the specific surface area were analyzed using a BELSORP-max (Microtrac BEL, Osaka, Japan). The N_2_ adsorption/desorption isotherms of the PACs were obtained at 77 K after degassing at 220 °C for 2 h under N_2_ gas. X-ray photoelectron spectroscopy (XPS) was conducted using a K-Alpha XPS instrument (Thermo Electron, Waltham, MA, USA). The high-resolution XPS spectra of C1s and O1s were obtained with a pass energy of 30 eV in 0.1 eV steps. Raman spectra of the PACs were obtained using an inVia Raman microspectrometer (Renishaw, Gloucestershire, UK). The excitation wavelength and spectral resolution were 514 nm and 4 cm^−1^, respectively. X-ray diffraction (XRD) patterns were obtained using a DB Advance X-ray diffractometer (Bruker, Germany) in the 2*θ* range of 3–89.14°. The zeta potential was analyzed with a suspension of 5 g/L PACs in a 0.01 M KCl aqueous solution in the pH range of 2–11 using a zeta potential analyzer (ZetaPlus, Malvern, UK). The total acidity, acidic groups, i.e., phenolic, lactonic, and carboxylic groups, and basicity were measured by standardized Boehm titration [39]. Briefly, a mixture of 0.2 g of a PAC and 20 mL of 0.1 N NaOH was prepared, shaken at 150 rpm for 48 h, and filtered through a 0.45 μm polyvinylidene fluoride (PVDF) filter. 20 mL of 0.1 N HCl was added to 10 mL of the filtrate, and then the mixture was back titrated with 0.05 N NaOH solution.

### 2.3. Experiment Procedures

The 1000 mg/L TC stock solution was prepared by dissolving 0.05411 g TC·HCl in DDIW, and the pH was adjusted to 7.0 ± 0.1 using 0.1 N aqueous solutions of HCl and NaOH. The total volume of DDIW, 0.1 N HCl, and 0.1 N NaOH was 50 mL.

Batch adsorption kinetic experiments were conducted in a 250 mL four-neck round flask placed in a heating mantle (MS3040, Misung Scientific Co., Ltd., Yangju, Korea) at 296.15, 310.15, and 318.15 K. The 0.08 g of the PACs were dispersed in 180 mL DDIW by mixing at 250 rpm and then, the 20 mL TC stock solution was introduced so that the initial TC concentration was 100 mg/L, to start adsorption. Samples were taken at 2, 5, 10, 15, 30, and 60 min, and filtered through a 0.45 μm PVDF filter.

The equilibrium adsorption isotherm experiments were performed in 50 mL amber glass vials. 0.05 g of the PACs were dispersed in 20 mL of DDIW in the vials. Then the TC stock solution and DDIW were introduced to obtain the final TC concentrations of 10–300 mg/L in the total solution volume of 40 mL. The vials were shaken in a water bath shaker (DS-250SW, Daewon Science, Inc., Cheongju-si, Korea) for 4 h at 150 rpm at 296.15, 310.15, and 318.15 K, and then, the mixtures were filtered through a 0.45 μm PVDF filter.

The pH did not change significantly with a value of 6.94 ± 0.25 for all experiments. The concentration of TC in the filtrates was measured using a high-performance liquid chromatography system (YL9100 Plus, Younglin, Anyang, Korea) with a C18 column (Eclipse Plus, 4.6 × 250 mm, 5 μm, Agilent, CA, USA). The mobile phase consisted of 0.01 M oxalic acid, acetonitrile, and methanol (70:20:10, *v*/*v*/*v*). The flow rate, oven temperature, detection wavelength, and injection volume were 1 mL/min, 30 °C, 360 nm, and 25 μL, respectively.

### 2.4. Modeling

The results of the adsorption kinetic experiments were fitted to a pseudo-first-order adsorption kinetic model (Equation (1)) [40], a pseudo-second-order adsorption kinetic model (Equation (2)) [41], an intra-particle diffusion model (Equation (3)) [42], and the Elovich equation (Equation (4)) [43].

(1) $$\frac{dq_{t}}{dt}=k_{a1}\left(q_{e1}-q_{t}\right)$$

(2) $$\frac{dq_{t}}{dt}=k_{a2}{\left(q_{e2}-q_{t}\right)}^{2}$$

(3) $$q_{t}=k_{id}t^{0.5}$$

(4) $$q_{t}=exp\left(\beta_{El}q_{t}\right)$$

Here, *q_t_* is the amount of TC adsorbed at time *t* (min) (mg/g), *q_e_* is the equilibrium adsorption amount (mg/g), *k_a_*_1_ is the pseudo-first-order adsorption rate constant (min^−1^), *k_a_*_2_ is the pseudo-second-order adsorption rate constant (g/mg‧min), *k_id_* is the rate constant of intra-particle diffusion, *α_El_* is the initial adsorption rate (mg/g‧min), and *β_El_* is a constant related to the surface coverage and activation energy of adsorption (g/mg).

The results of the equilibrium adsorption isotherm experiments were described using Langmuir (Equation (5)) [44], Freundlich (Equation (6)) [45], and Temkin (Equation (7)) [46] isotherms.

(5) $$q_{e}=q_{max}\frac{K_{L}C_{e}}{1+K_{L}C_{e}}$$

(6) $$q_{e}=K_{F}C_{e}{}^{1/n}$$

(7) $$q_{e}=\frac{RT}{b_{T}}lnA_{T}C_{e}$$

Here, *q_e_* is the equilibrium adsorption amount (mg/g), *q_max_* is the maximum adsorption capacity (mg/g), *C_e_* is the equilibrium concentration of TC (mg/L), *K_L_* is the Langmuir adsorption constant (L/mg), *K_F_* is the Freundlich constant ((mg/g)‧(L/mg)^1/n^), *1/n* is a constant related to the adsorption intensity, *R* is the gas constant (8.314 J/mol‧K), *T* is the absolute temperature (K), *b_T_* is the Tempkin constant associated with the heat of adsorption (J/mol), and *A_T_* is the Tempkin equilibrium constant (L/mg).

## 3. Results

### 3.1. Effects of the Thermal Treatment Temperature on TC Adsorption

It was found that the TC adsorption onto PAC was enhanced from 124.2 to 178.4 mg/g as the thermal treatment temperature was increased from 500 to 800 °C (Figure S1). Therefore, the properties and TC adsorption of PAC and PAC800 were investigated in detail. Meanwhile, Figure S1 shows that TC adsorption was suppressed when the PAC was subjected to 900 °C. It is thought that the increase in the TC adsorption with increasing treatment temperature is attributed to the increase in pores and specific surface area as demonstrated in other studies [47,48]. However, excessive temperature can cause the destruction of pore walls to increase larger pores [47] and can enhance gasification reaction to reduce pores and surface area [49]. These can lead to the reduction of adsorption.

### 3.2. Characterization

#### 3.2.1. N_2_ Adsorption/Desorption

**Figure 1:**
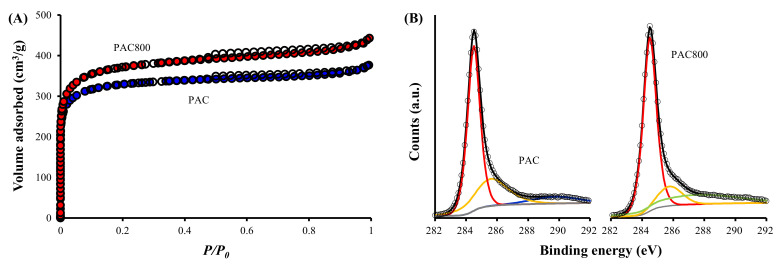
(**A**) N_2_ adsorption/desorption and (**B**) C1s XPS spectra of PAC and PAC800.

The specific surface area (*S_BET_*), total pore volume (*V_p_*), and average pore diameter (*d_p_*) increased after thermal treatment at 800 °C (Table 1). Both PAC and PAC800 showed a combination of Type I(b) and Type II isotherms with an H4 hysteresis loop, suggesting that their pores consist of micro-, meso-, and macropores [50,51] (Figure 1A). However, it is also indicated that the pores of PAC800 have a wider size distribution, as supported by the distribution of mesopores and micropores presented in Figure S2A,B. It is also observed that PAC800 has smaller meso- and micropores than PAC. Therefore, the higher *d_p_* of PAC800 compared to PAC is attributable to the larger amount of macropores in PAC800 than in PAC. This suggests that the thermal treatment of the PAC increased the number of macropores, as well as micro- and mesopores.

#### 3.2.2. XPS

The fractions of C and O were 87.36% and 12.64% for PAC, and 88.52% and 11.48% for PAC800, respectively. Therefore, the ratio of C to O (C/O) increased from 6.91 to 7.72, respectively. The C1s spectrum of PAC showed characteristic peaks at 284.5, 285.6, and 289.7 eV, which are assigned to graphitic C-C, C-O, and O-C=O, respectively [52] (Table S2, Figure 1B). After thermal treatment, the graphitic C-C peak increased from 62.9% for PAC to 66.0% for PAC800. However, the C-O peak decreased greatly from 28.0% for PAC to 13.0% for PAC800, and the O-C=O peak of PAC disappeared. Instead, a new peak at 287.5 eV, representing the carbonyl groups (C=O) of quinone and pyrone [52], appeared with a substantial fraction of 21.0%. The changes in the XPS spectra are attributable to the thermal treatment. It was reported that the thermal treatment of activated carbons and biochar under inert gas results in a decrease of hydrophilic groups, such as O-C=O, and an increase of hydrophobic groups, such as C=O [53], in addition to graphitic C-C [28].

Meanwhile, the full width at half-maximum (FWHM) of the graphitic C-C peak did not show a notable difference. However, the FWHM of C-O significantly decreased from 2.54 for PAC to 1.88 for PAC800, indicating enhanced crystallinity. It was also previously reported that the crystallinity of activated carbon and biochar was improved via thermal treatment [28,54].

#### 3.2.3. Raman Spectroscopy

**Figure 2:**
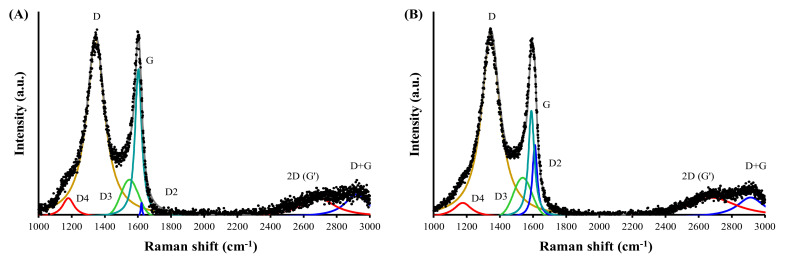
Raman spectra of (**A**) PAC and (**B**) PAC800.

The Raman spectra were deconvoluted to several bands at 1176–1180, 1345, 1538–1532, 1590–1598, and 1611–1620 cm^−1^, which are assigned to D4, D, D3 (D’), G, and D2 (D’) bands, respectively (Figure 2, Table S3).

The D4 band is associated with disordered graphitic lattice, polyenes, and ionic impurities, and it did not show a notable change after thermal treatment. The bands of D, D3 (D’), G, and D2 (D’) are associated with disordered graphitic lattice induced by the *sp^3^* hybridization such as graphene layer edges, amorphous carbon, ideal graphitic lattice with *sp^2^*-C atoms, and disordered graphitic lattice in surface graphene layers, respectively [55].

The thermal treatment of PAC resulted in a slight increase of the D band as well as a large increase of the G band, which resulted in a decrease of the ratio of the intensity of the D band to that of the G band, i.e., *I_D_/I_G_*, from 5.4 in PAC to 3.0 in PAC800. This indicates a decrease in the structural defects of graphitic structures [56] after thermal treatment. The significant decrease of the D3 band indicates a decrease in amorphous carbon and increased crystallinity by the thermal treatment. A great decrease of the D2 band in PAC800, compared to PAC, indicates a significant decrease in graphene surfaces via the stacking of them to form graphitic structures. The ratio of the intensity of the D band to that of the D2 band, i.e., *I_D_/I_D2_*, of PAC was 9.8, indicating that the defects were associated with hopping defects, which are formed by the deformation of carbon bonds. On the other hand, the D2 band of PAC800 was negligible to result in an extraordinary value of *I_D_/I_D2_* (111.1), suggesting that the defects were associated with charged impurities, i.e., atoms and/or molecules, adsorbed onto graphene layers [57].

The Raman spectra also showed clear bands at 2684 and 2913–2919 cm^−1^, which are assigned to 2D (G’) and D + G (D + D’) bands, respectively [55,58]. They are associated with single-layer graphene and a combination of the D and G bands, respectively [55] (Figure 2, Table S3). The 2D (G’) band was significantly decreased and, as a result, the ratio of the intensity of the 2D band to that of the G band, i.e., *I_2D_/I_G_*, decreased from 1.6 for PAC to 0.6 for PAC800. This suggests that the number of single graphene layers was decreased by the thermal treatment, which is in agreement with the decrease of the D2 (D’) band and the increase of *I_D_/I_D2_*.

Collectively, the thermal treatment of PAC resulted in a more crystalline and more stacked graphitic structure with fewer defects.

#### 3.2.4. XRD, Zeta Potential, and Titration

The results of titration showed higher basicity and lower acidity and acidic groups in PAC800, compared to PAC (Table 2). This agrees with the results of XPS (Table S2 and Figure 1B), supporting the thermal decomposition of acidic groups [53].

The XRD patterns are provided in Figure S3A. The broad peak at *2θ* ≈ 16°, which is assigned to (101) cellulose [59], was found for both PAC and PAC800, without a significant difference. A broad peak of the (100) plane of the graphitic structures was found at *2θ* ≈ 43.5°, without a notable difference between PAC and PAC800. However, it was shown that a peak representing the (002) interlayer spacing of graphitic structures (*2θ* ≈ 26° and 30°) [60] was more intensive and sharper for PAC800 than PAC. This indicates that there are more crystalline and ordered graphite structures and increased *sp^2^*-C in PAC800 than in PAC [61], as also indicated by the Raman spectroscopy results.

The zeta potentials of PAC and PAC800 are presented in Figure S3B, showing that PAC800 was more positive than PAC under the same pH. The point of zero charges (pH_pzc_) values of PAC and PAC800 were around 2 and 8, respectively. This indicates that the attraction of anions, therefore physisorption, to PAC800 is stronger than to PAC. Also, the results in Figure S3B are attributable to the decomposition of acidic groups, as also suggested in Tables S2 and S3 and Figure 1B, which is responsible for the negative charge of carbonaceous materials [62].

### 3.3. Adsorption Kinetics

**Figure 3:**
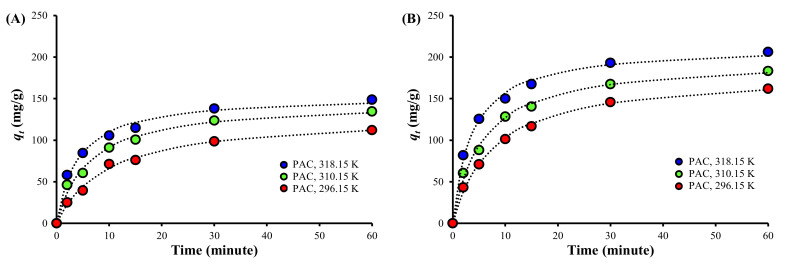
The experimental results of TC adsorption kinetics of (**A**) PAC and (**B**) PAC800 at different temperature, with the results of the fit to the pseudo second-order adsorption kinetic model (dotted lines).

Figure 3 shows that the TC adsorption amount was higher for PAC800 than for PAC at the same time and temperature. It was also shown that the adsorption was enhanced with increasing temperature for both PAC and PAC800, suggesting that the TC adsorption onto PAC and PAC800 is endothermic.

Table 3 shows that the pseudo-second-order model was the best fit for all of the experimental results, as represented by the dotted lines in Figure 3. The pseudo-first-order model estimated equilibrium, as previously reported [63,64], and provided poor fits to the results in this study (Figure S4). The intraparticle diffusion model was also a poor fit to all of the experiment results (Figure S5), indicating that the adsorption was not governed by the diffusion of TC through the pores of PAC or PAC800, but by the diffusion across the liquid film on the adsorbents [65]. However, the Elovich equation described the adsorption well with high correlation coefficients (*r^2^*) (Figure S6), which indicates that the adsorption is the result of a combination of chemisorption, bulk diffusion, and surface diffusion on energetically heterogeneous surfaces and that the adsorption rate decreases with an increasing surface coverage [66].

Table 3 also shows that the adsorption rate, i.e., *k_a2_*, was reduced, while the equilibrium adsorption amount, i.e., *q_e2_*, was enhanced after thermal treatment at 800 °C. This can be attributed to the increase of smaller pores in PAC800 compared to PAC (Figure S2A,B), which would limit the mass transfer of TC because the adsorption onto porous materials is generally controlled by pore diffusion [67]. On the contrary, the *α_El_* of PAC800 was higher than that of PAC at the same temperature, indicating that the initial TC adsorption was faster for PAC800 than for PAC [66]. Therefore, it is thought that the reduction of the adsorption rate with increasing amount of TC on the surfaces was more significant for PAC800 than for PAC, considering the lower *k_a2_* of PAC800 compared to PAC.

### 3.4. Adsorption Isotherm

**Figure 4:**
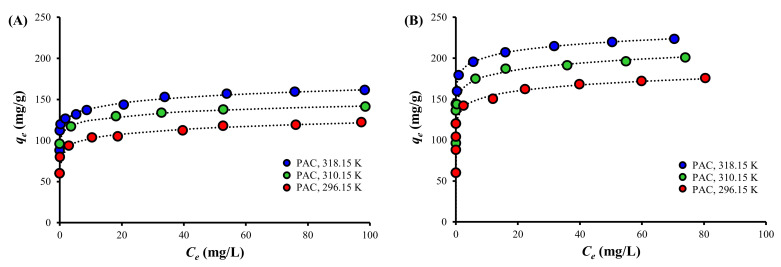
The experimental results of TC adsorption isotherm by (**A**) PAC and (**B**) PAC800 at different temperature and the fit of Freundlich isotherm model (dotted lines).

The results of the fits of various isotherms are given in Table 4, while the experimental results and the results of the best fit, i.e., Freundlich isotherm, are presented in Figure 4. The Langmuir isotherm was a poor fit, whereas the Freundlich isotherm was a good fit for all of the experimental results. This suggests that the TC adsorption onto both PAC and PAC800 is not limited to monolayer formation and is heterogeneous, where the adsorption enthalpy decreases with increasing surface coverage [68], as indicated by the good fit of the pseudo-second-order model (Table 3).

The *K_F_* value, which is indicative of the adsorption capacity, increased with increasing temperature. The values of *1/n* were in the range of 0.053–0.076, suggesting that the TC adsorption onto both PAC and PAC800 is favorable, nearly irreversible, and includes significant involvement of chemisorption. The *1/n* value is the intensity of adsorption, which indicates whether the adsorption is favorable (0 < *1/n* < 1), unfavorable (*1/n* > 1), or irreversible (*1/n* = 0) [68,69].

The Temkin isotherm was a poor fit although heterogeneity was also assumed. However, a linear and a logarithmic decrease in the adsorption heat is well described by the Temkin and Freundlich isotherms, respectively [69]. Therefore, it is suggested that the adsorption energy decreases logarithmically with an increasing surface coverage of both PAC and PAC800.

### 3.5. Thermodynamics

The activation energy (*E_a_*) of adsorption was calculated using an Arrhenius-type relationship between the rate constant of the pseudo-second-order model, which was the best fit (*k_a2_*) (Equation (2)) [65], while the Gibbs’ free energy change (Δ*G*^0^) and changes of enthalpy (Δ*H*^0^) and entropy (Δ*S*^0^) were calculated using Equations (8)–(10) [68] (Table 5, Figure S7).

(8) $$k_{a2}=k_{a2}\prime exp\left(\frac{E_{a}}{RT}\right)\;$$

(9) $$\Delta G^{0}=-RTln\left(K^{0}\right)$$

(10) $$ln\left(K^{0}\right)=-\frac{\Delta H^{0}}{RT}+\frac{\Delta S^{0}}{R}$$

Here, *E_a_* is the activation energy (J/mol), *R* is the gas constant (8.31446 J/mol⋅K), *T* is the temperature (K), and *K^o^* is the equilibrium constant calculated via the correlation of the adsorbate mass on the adsorbent surface (*m_s_*) per that in the liquid phase (*m_e_*) (Figure S8).

The *E_a_* values of PAC and PAC800 were 23.7 and 19.6 kJ/mol, respectively, indicating a lower energy barrier of TC adsorption onto PAC800 than PAC, which may be responsible for the improved TC adsorption after the thermal treatment.

The Δ*G*^0^ value decreased as the temperature was increased, indicating that the TC adsorption is more spontaneous at high temperatures for both PACs, also supporting the endothermic adsorption, as evidenced before (Table 3 and Table 4, Figure 3 and Figure 4). The Δ*G*^0^ value was significantly lower for PAC800 than for PAC at the same temperature, indicating a higher affinity of PAC800 to TC than PAC.

Both PAC and PAC800 showed positive Δ*H*^0^ values, indicating the endothermic nature of the adsorption, and positive Δ*S*^0^ values, suggesting the increase of the randomness during the adsorption, which may enhance the endothermic property [70]. The Δ*H*^0^ values of PAC and PAC800 were over 40 kJ/mol, indicating the dominance of chemisorption with strong binding between TC and the PACs, rather than physisorption. This is in agreement with the low *1/n* calculated by the Freundlich isotherm, indicating nearly irreversible adsorption (Table 5). However, it decreased after the thermal treatment, which suggests that physisorption was more involved and the TC adsorption was weaker for PAC800 than for PAC [70].

### 3.6. Factors Affecting TC Adsorption and the Potentials in Real Applications

It should be noted that the TC adsorption per unit surface area, i.e., *q_max_* divided by *S_BET_*, was higher for PAC800 than for PAC at the same temperature. The values obtained were 0.099 and 0.126 mg/m^2^ at 296.15 K, 0.114 and 0.143 mg/m^2^ at 310.15 K, and 0.129 and 0.159 mg/m^2^ at 318.15 K, for PAC and PAC800, respectively. This strongly suggests that the improved TC adsorption of PAC800 was not only attributed to the increase of *S_BET_* but significantly to the changes in the surface properties of PAC after the thermal treatment.

It is known that electrostatic attraction, hydrophobic interaction, and π-π EDA interactions are responsible for the adsorption of organic compounds onto carbonaceous adsorbents, as mentioned above, and that they are largely dependent on the characteristics of the adsorbent [38].

Firstly, it is thought that the enhanced electrostatic attraction is one of the reasons for the improved TC adsorption onto PAC800 than PAC. TC has three (3) pK_a_s, 3.3, 7.7, and 9.7, and TC^0^ was dominant with lower amounts of TC^−^ considering the pH during the adsorption in this study, i.e., 6.94 ± 0.25 [71]. Figure S3B shows that the zeta potential of PAC800 was substantially higher than that of PAC over a wide range of pH with values of 6.22 ± 1.34 and −39.62 ± 4.6 eV at a pH of 7.0 for PAC800 and PAC, respectively. Therefore, it seems certain that the enhanced electrostatic attraction of PAC800 contributed to the higher adsorption of TC compared to PAC. This is supported by the lower Δ*H* value of PAC800 than PAC, indicating that physisorption, i.e., electrostatic attraction, is relatively more responsible for the TC adsorption onto PAC800 than onto PAC.

Secondly, it does not seem that enhanced hydrophobic interaction contributed to the higher TC adsorption capacity of PAC800 than PAC. The increased hydrophobic nature in PAC800 was evidenced by an increase of more crystalline graphitic C-C and C=O (Tables S2 and S3, Figure S3), due to the thermal decomposition of hydrophilic groups [53]. However, TC is hydrophilic with a log *K_ow_* of −1.3 [11].

Thirdly, it seems reasonable that π-π EDA interactions played an important role in the improved TC adsorption of PAC800. The increase of the graphitic C-C with enhanced crystallinity as well as oxygen-containing functional groups, i.e., C=O, after the thermal treatment was evidenced by XPS, Raman spectroscopy, and XRD pattern analyses (Tables S2 and S3, Figure S3). They are effective π-electron donors, improving the adsorption of organic molecules [38]. It was reported that graphene oxides, enriched with oxygen-containing functional groups, showed very high acetaminophen adsorption capacity [72].

Collectively, it seems reasonable that the enhanced TC adsorption onto PAC800, compared to PAC, is attributable to the increased surface area as well as the enhanced electrostatic attraction and π-π EDA interactions via higher surface charge and more crystalline graphitic C-C and C=O. It was also found by, the theoretical calculations of the density functional theory, that there are strong π-π EDA interactions between electron-deficient conjugated enone structures of TC molecules and electron-rich graphitic surfaces of an AC [73]. In addition, the results of the molecular surface electrostatic potential analysis showed that the TC adsorption onto an AC is via electrostatic interactions between the graphitic AC surface and the electrophilic groups of TC and π-π EDA interactions between TC ring planes and delocalized π electrons in graphitic structures [74].

Meanwhile, the performance of the PACs in this study in real waters must be discussed to evaluate the feasibility in practical applications, because the adsorption of pollutants in water is largely dependent on the water matrix [75]. The components often detected in water and wastewater are cations, such as Na^+^, K^+^, Ca^2+^, NH_4_^+^, and Mg^2+^, anions, such as NO_3_^−^, PO_4__3_^−^, SO_4__2_^−^, and HCO_3_^−^, and natural organic matter (NOM) [76]. It is thought that the inorganic ions would not significantly affect the TC adsorption by PAC800 because they are not readily adsorbed onto ACs [77,78], resulting in negligible competition. On the contrary, the effect of NOM must be evaluated before real applications. NOM is slightly hydrophobic and can compete with TC for the adsorption sites on ACs [78], seriously affecting the adsorption. However, adsorption is not only affected by the water matrix but also by the property of the adsorbent significantly, such as functional groups, graphitic structures, and pore structures [28,37,78]. Therefore, it is thought that the matrix effects would be suppressed via the proper modification of ACs.

## 4. Conclusions

In this study, a coconut shell-based PAC was thermally modified to improve TC adsorption. The results showed that the PAC subjected to the thermal treatment at 800 °C, i.e., PAC800, showed the best performance.

The N_2_ adsorption/desorption isotherms showed increased SBET for PAC800 than for PAC. The results of XPS, Raman spectroscopy, XRD, and zeta potential analyses demonstrated an increase of C content, C=O (quinone and pyrone), and surface charge as well as a decrease of acidic groups. It was also shown that the structural defects of the graphitic structures in PAC were changed into defects with charged impurities in PAC800. In addition, the graphitic structures of PAC800 were crystalline and more stacked than PAC.

The TC adsorption capacity of PAC800 was significantly higher than that of PAC, which is attributable to the increased SBET, electrostatic attraction, and π-π EDA interactions, considering the properties of PAC and PAC800. The TC adsorption onto both PAC and PAC800 was endothermic with positive Δ*H*^0^ values and spontaneous with negative Δ*G*^0^ values. The good fits of the pseudo-second-order model, Elovich equation, and Freundlich isotherm to the experimental results suggested that the TC adsorption onto both PAC and PAC800 is heterogeneous, which is affected by surface coverage and a combination of chemisorption, diffusion, electrostatic attraction, and π-π EDA interactions. However, PAC800 showed lower *E_a_* and Δ*H*^0^ values compared to PAC, suggesting that more physisorption is involved and that the adsorption is more reversible for PAC800 than PAC.

The results of this study strongly suggest that the properties including the adsorption capacity of micropollutants as well as the adsorption mechanisms of ACs can be significantly changed by simple thermal treatment. In addition, the changes in the characteristics of PAC, which may positively affect the TC adsorption, would provide valuable information about the strategy of design and regeneration of carbonaceous adsorbents for micropollutant removal.

## Figures and Tables

**Table 1:** Results of N_2_ adsorption/desorption isotherms.

	*S_BET_* (m^2^/g)	*V_p_* (m^3^/g)	*d_p_* (Average, nm)
PAC	1252.9	0.581	1.855
PAC800	1410.8	0.681	1.930

**Table 2:** Results of titration (mmol/mg).

	Basicity	Acidity	Phenolic Groups	Lactonic Groups	Carboxylic Groups
PAC	2.086	0.890	0.531	0.246	0.113
PAC800	2.736	0.174	0.115	0.059	ND

**Table 3:** Results of adsorption kinetics.

Model	Constants	PAC			PAC800		
		296.15 K	300.15 K	318.15 K	296.15 K	300.15 K	318.15 K
Pseudo first order	*k_a_*_1_ (min^−1^)	0.094	0.130	0.182	0.110	0.141	0.205
	*q_e_*_1_ (mg/g)	108.4	127.8	136.9	155.0	173.0	190.4
	*r* ^2^	0.986	0.969	0.957	0.983	0.977	0.966
Pseudo second order	*k_a_*_2_ (×10^−3^ g/mg․min)	0.80	1.12	1.59	0.72	0.90	1.29
	*q_e_*_2_ (mg/g)	129.9	146.5	154.3	181.3	198.0	214.0
	*r* ^2^	0.993	0.989	0.992	0.998	0.995	0.996
Intraparticle diffusion	*k_id_* (mg/g·min^−0.5^)	16.8	21.3	24.2	25.0	29.3	32.5
	*r^2^*	0.916	0.829	0.727	0.886	0.807	0.676
Elovich equation	*α_El_* (mg/g·min)	31.0	65.2	120.2	57.6	93.9	199.5
	*β_El_* (g/mg)	0.0371	0.0358	0.0364	0.0274	0.0264	0.0269
	*r* ^2^	0.980	0.979	0.952	0.994	0.985	0.985

**Table 4:** Results of adsorption isotherm.

		PAC			PAC800		
		296.15 K	310.15 K	318.15 K	296.15 K	310.15 K	318.15 K
Langmuir	*q_max_* (mg/g)	117.4	138.5	154.7	169.6	199.5	224.5
	*K_L_* (L/mg)	1.151	1.426	1.852	1.846	1.073	1.196
	*r* ^2^	0.778	0.902	0.714	0.733	0.829	0.920
Freundlich	*K_F_* ((mg/g)(L/g)^1/*n*^)	85.8	109.3	119.5	132.1	154.9	178.6
	*1/n*	0.076	0.057	0.066	0.064	0.061	0.053
	*r* ^2^	0.981	0.994	0.983	0.969	0.989	0.999
Temkin	*b_T_* (J/mol)	1.07 × 10^−3^	1.26 × 10^−3^	1.41 × 10^−3^	1.47 × 10^−3^	2.28 × 10^−3^	2.44 × 10^−3^
	*A_T_* (L/mg)	7.28 × 10^16^	8.52 × 10^16^	1.81 × 10^16^	6.12 × 10^16^	1.50 × 10^13^	1.78 × 10^13^
	*r* ^2^	0.529	0.663	0.606	0.601	0.800	0.813

**Table 5:** Thermodynamic parameters of TC adsorption by PAC and PAC800.

	PAC			PAC800		
	296.15 K	310.15 K	318.15 K	296.15 K	310.15 K	318.15 K
*E_a_* (J/mol)	23.7			19.6		
Δ*G*^0^ (kJ/mol)	−34.23	−47.89	−51.47	−41.17	−48.91	−51.28
Δ*H*^0^ (kJ/mol)	196.7			98.5		
Δ*S*^0^ (kJ/mol·K)	0.780			0.473		

## Supplementary Materials

The following are available online at https://www.mdpi.com/article/10.3390/ijerph192113741/s1, Table S1. Chemical structure and properties of tetracycline; Table S2. Results of high-resolution XPS of C1s; Table S3. Results of Raman spectroscopy; Figure S1. TC adsorption of the PACs treated at different temperature; Figure S2. (A) The distribution of mesopores, and (B) the distribution of micropores of PAC and PAC800; Figure S3. (A) XRD patterns and (B) zeta potential of PAC and PAC800; Figure S4. TC adsorption kinetics of (A) PAC and (B) PAC800 at different temperature, with the results of the fit to the pseudo first-order adsorption kinetic model (dotted lines); Figure S5. TC adsorption kinetics of (A) PAC and (B) PAC800 at different temperature, with the results of the fit to the intraparticle diffusion model (dotted lines); Figure S6. TC adsorption kinetics of (A) PAC and (B) PAC800 at different temperature, with the results of the fit to the Elovich equation (dotted lines); Figure S7. The correlations of temperature and (A) *k_a_*_2_ and (B) *K*_0_; Figure S8. The correlations between *m_s_*/*m_e_* and *m_s_* of (A) PAC and (B) PAC800 at different temperature. References [1,79] are cited in the Supplementary Materials.
