# Supplementary material for: Enhanced Adsorption of Tetracycline by Thermal Modification of Coconut Shell-Based Activated Carbon
Source: Int J Environ Res Public Health. 2022 Oct 22;19(21):13741. doi: 10.3390/ijerph192113741 (PMC9655672; doi:10.3390/ijerph192113741)
Supplement: Supplementary file 1 [file ijerph-19-13741-s001.zip › ijerph-1905426-supplementary.pdf]

Supplementary information

Enhanced Adsorption of Tetracycline by Thermal Modification of Coconut Shell-Based Activated Carbon

Do-Gun Kim [1], Shinnee Boldbaatar [1] and Seok-Oh Ko [2,*]

[1] Department of Environmental Engineering, Sunchon National University, 255 Jungang-ro, Suncheon 57922, Korea
[2] Department of Civil Engineering, Kyung Hee University, 1732, Deakyungdaero, Yongin 17104, Korea
* Correspondence: soko@khu.ac.kr; Tel.: +82-31-201-2999

Table S1. Chemical structure and properties of tetracycline.

Chemical structure	CAS No.	Molecular formula	Molecular weight (g/mol)	Water solubility (mol/L)	Melting point (°C)	log K_{ow}	pK_a	Molar absorption at 254 nm (m^2/mol)
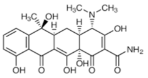	60-54-8	$C_{22}H_{24}N_2O_8$	480.9	0.041	172.5	-1.25	3.2, 7.7, 9.6	1203

Reference:
Marzbali MH, Esmaieli M, Abolghasemi H, Marzbali MH. 2016. Tetracycline adsorption by H_3PO_4-activated carbon produced from apricot nut shells: A batch study. Process Safety and Environmental Protection 102, 700–709.
Xu L, Zhang H, Xiong P, Zhu Q, LiaoC, Jiang G. 2021. Occurrence, fate, and risk assessment of typical tetracycline antibiotics in the aquatic environment: A review. Sci Total Environ 753, 141975.

Table S2. Results of high-resolution XPS of C1s.

		**Graphitic C-C**	**C-O**	**C=O**	**O-C=O**
PAC	Position (eV)	284.5	285.6	-	289.7
	Fraction (%)	62.9	28.0	-	9.0

	FWHM (eV)	0.95	2.54	-	3.59
PAC800	Position (eV)	284.5	285.8	287.5	-
	Fraction (%)	66.0	13.0	21.0	-
	FWHM (eV)	0.99	1.88	6.34	-

Table S3. Results of Raman spectroscopy.

		D4 (I)	D (D1)	D3 (D'', A)	G	D2 (D')	2D (G')	D+G (D+D')
PAC	Center (eV)	1,176	1,345	1,538	1,590	1,611	2,684	2,913
	Fraction (%)	3.9	51.1	7.3	9.5	5.2	14.9	8.1
	FWHM (eV)	129.4	133.0	131.3	42.9	34.7	366.4	197.0
PAC800	Center (eV)	1,180	1,345	1,532	1,598	1,620	2,684	2,919
	Fraction (%)	3.6	52.9	5.9	17.5	0.5	10.8	8.7
	FWHM (eV)	85.5	130.7	130.6	49.8	12.3	246.1	173.3

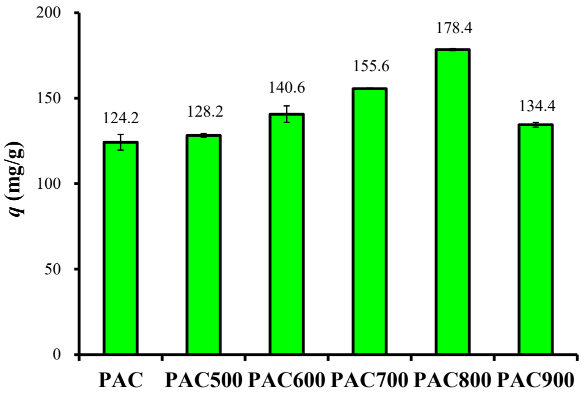

Figure S1. TC adsorption of the PACs treated at different temperature.

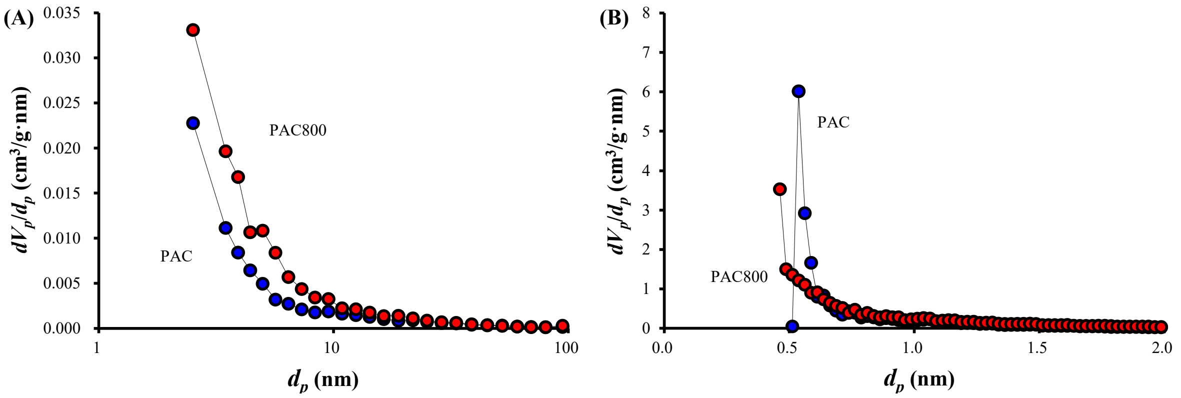

Figure S2. (A) The distribution of mesopores, and (B) the distribution of micropores of PAC and PAC800.

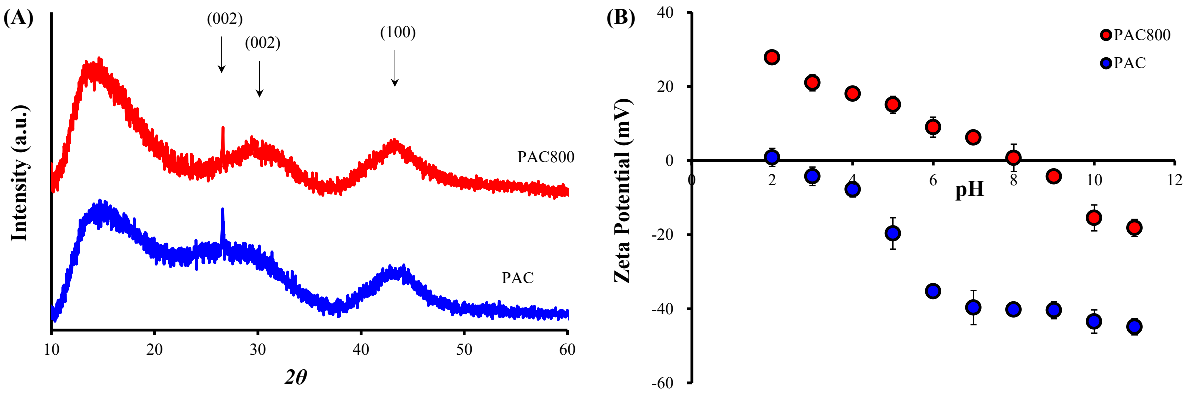

Figure S3. (A) XRD patterns and (B) zeta potential of PAC and PAC800.

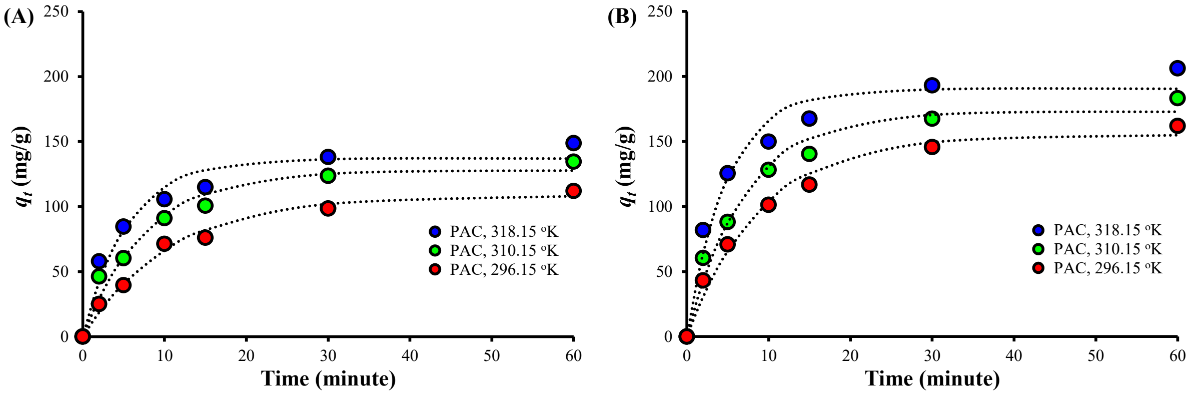

Figure S4. TC adsorption kinetics of (A) PAC and (B) PAC800 at different temperature, with the results of the fit to the pseudo first-order adsorption kinetic model (dotted lines).

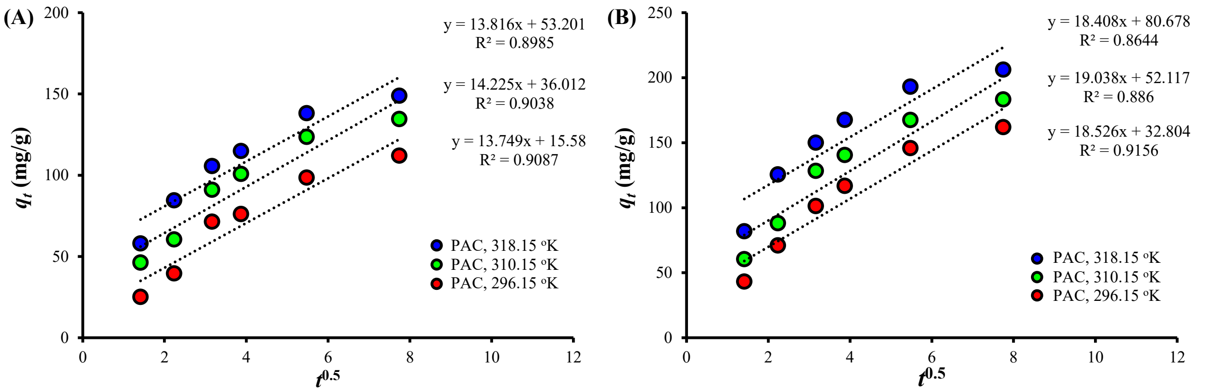

Figure S5. TC adsorption kinetics of (A) PAC and (B) PAC800 at different temperature, with the results of the fit to the intraparticle diffusion model (dotted lines).

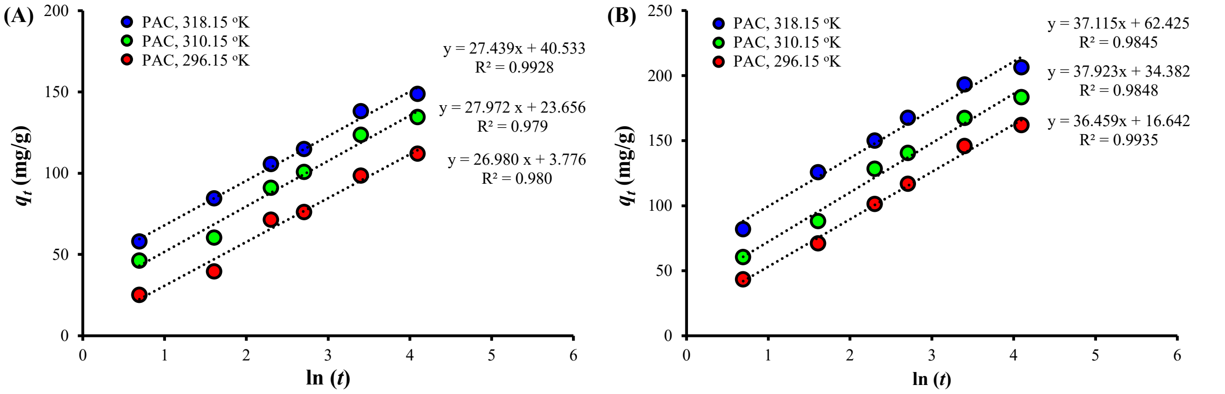

Figure S6. TC adsorption kinetics of (A) PAC and (B) PAC800 at different temperature, with the results of the fit to the Elovich equation (dotted lines).

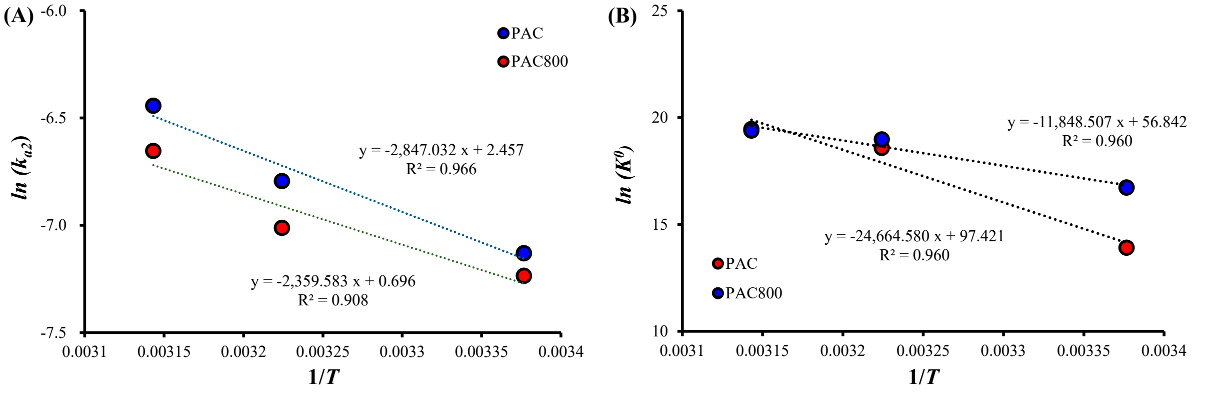

Figure S7. The correlations of temperature and (A) k_{a2} and (B) K_0.

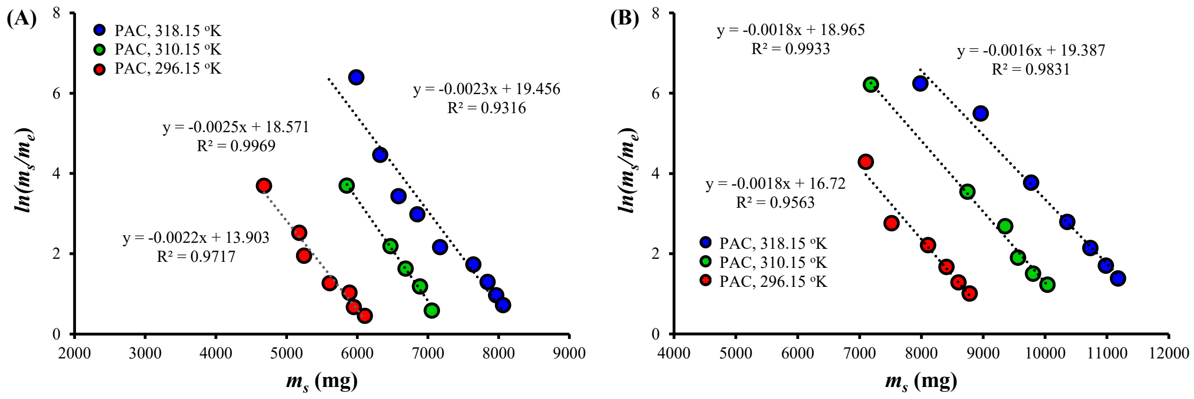

Figure S8. The correlations between m_s/m_e and m_s of (A) PAC and (B) PAC800 at different temperature.
